# Supplementary material for: Advanced stage presentation and its determinant factors among colorectal cancer patients in Amhara regional state Referral Hospitals, Northwest Ethiopia
Source: PLoS One. 2022 Oct 7;17(10):e0273692. doi: 10.1371/journal.pone.0273692 (PMC9543633; doi:10.1371/journal.pone.0273692)
Supplement: Attachment — Submitted filename: Response to reviewers comment.docx [file pone.0273692.s003.docx]

**Manuscript ID: PONE-D-22-07229R1**

**Topic: Advanced stage presentation and its determinant factors among colorectal cancer patients in Northwest Amhara Regional State Referral Hospitals of Ethiopia**

**Dear Editors and reviewers;**

We sincerely appreciate the valuable comments and suggestions from the reviewers and editors side. The suggestions and comments have been closely followed and revisions have been made accordingly. The following are the questions extracted from the reviewers’ comments along with our summarized responses. Thank you very much for your constructive comments. We tried to inculcate your comments and questions as described below. The changes are attached with. Much correction has been made through the document.

Editor comments

**Editor’s comment #01**: There is a questionable difference between the manuscript-mentioned study period and the dates available in the datasheet.

**Author’s response:**

**Thank you very much for your constructive comments**: Your concern is quite right. Sorry for the inconvenience. But there is a difference in Ethiopian calendar (E.C) and Gregorian calendar (G.C). On the dataset, time of study participants enrollment was by Ethiopian calendar since the data is registered in E.C. But the time in the manuscript was written by G.C. By adding 8 years (from January to August) on 2009 E.C equals with 2017 in G.C and adding 7 years (from September to December) on 2013 E.C equals 2020 G.C. This 8 and 7 years difference is due to the new year of January and September in G.C and E.C respectively. During analysis we changed Ethiopian calendar to Gregorian calendar. Kindly understand it in such a manner.

**Editor’s comment #02**: A native language edit of manuscript is recommended.

**Author’s response:** Thank you very much for the comment: sure! The manuscript has been reviewed by different personnel and many editorial and grammatical issues have been addressed.

**Reviewer #01**

Reviewer 1 comments and suggestion #01: The authors tried to address my comments and suggestions in this revision and I can say they were successful to some extent. Most of the scientific comments have been addressed. One major issue still remains that is the several grammatical and typo errors and the poor language of this manuscript. The prepared draft needs a revision by a native editor or English expert and the language and structure of manuscript should be revised in this regard, otherwise it may jeopardize the efforts of the authors.

**Author’s response:**

Thank you very much for your critical comments for the improvement of the manuscript. The manuscript has been reviewed by different personnel and many editorial and grammatical issues have been addressed.

**Reviewer #02**

Reviewer 2 comments and suggestion #01: Thank you for implementing the comments. Manuscript will require thorough proofreading to meet the required standards of language.

**Author’s response:** Thank you very much for your critical comments for the improvement of the manuscript. The manuscript has been reviewed by different personnel and many editorial and grammatical issues have been addressed.

**Reviewer #03**

Reviewer 3 comments and suggestion #01: In this manuscript, the authors aimed to assess stage of presentation and its contributing factors among colorectal cancer patients in Amhara regional state of Ethiopia. The manuscript was reviewed once in the previous round and revised by the authors. I reviewed previous comments and authors' responses, and most of them were adequate. Just to mention some English fluency issues still were in the manuscript. A copyediting by an expert is recommended. As the academic editor requested uploading the minimal anonym zed dataset, the authors uploaded the minimal data used to extract the report in the form of a datasheet in the supplements. As I reviewed the datasheet two issues raised some questions. First, the number of participants was 367 in the datasheet, which was also mentioned in the last sentence of the "Sample size determination and procedure" subheading in "Materials and Methods", but as we can see in all other parts of the manuscript the total number of participants was reported 376. Another issue is that in the "Study design, Period, and settings" part, it was mentioned that "patients enrolled from January 1, 2017, to December 31, 2020", but in the datasheet, "Date_of_last_visit" is from 2009 to 2013. As it seems from this evidence the participants' recruitments were not from 2017 to 2020. I would like to receive the authors' response in this regard.

**Author’s response:**

Thank you very much for your critical comments. The manuscript has been reviewed by different personnel and many editorial and grammatical issues have been addressed. You are right about the sample size difference but this is due to type errors, the exact number of sample size is 367.

**The other concern that you raise is about enrollments of study participants**:

Your concern is quite right. Sorry for the inconvenience. But there is a difference in Ethiopian calendar (E.C) and Gregorian calendar (G.C). On the datasheet time of study participants enrollment was by Ethiopian calendar since the data is registered in E.C. But the time in the manuscript was written by G.C. By adding 8 years (from January to August) on 2009 E.C equals with 2017 in G.C and adding 7 years (from September to December) on 2013 E.C equals 2020 G.C. This 8 and 7 years difference is due to the new year of January and September in G.C and E.C respectively. During analysis we changed Ethiopian calendar to Gregorian calendar. Kindly understand it in such a manner.
