# Supplementary material for: Advanced stage presentation and its determinant factors among colorectal cancer patients in Amhara regional state Referral Hospitals, Northwest Ethiopia
Source: PLoS One. 2022 Oct 7;17(10):e0273692. doi: 10.1371/journal.pone.0273692 (PMC9543633; doi:10.1371/journal.pone.0273692)
Supplement: Attachment — Submitted filename: Response to reviewers comment .docx [file pone.0273692.s004.docx]

10/08/2022

Dear Academic Editor,
PLOS ONE

Subject: Submission of **revised manuscript entitled as “Advanced stage presentation and its determinant factors among colorectal cancer patients in Northwest Amhara Regional State Referral Hospitals of Ethiopia (**PONE-D-22-07229R2**).**

Dear, Negar Rezaei, M.D., Ph.D, thank you for your email enclosing the Editorial member’s and the reviewer’s comments. We have carefully revised the manuscript and incorporated their comments accordingly. Our responses are given in point-by-point response below.

We hope the revised version is suitable for publication and look forward to hearing from you in due courses.

Sincerely

Mulugeta Wassie.

**Point by point responses to Editorial Board Member’s and Reviewers’ comments**

Editor comments

**Editor’s comment #01**: Please consider the reviewer 3 comments

**Authors’ response:** Thank very much for your suggestions. We have incorporated all the comments raised by reviewer 3 in the manuscript

**Reviewers’ comments:**

**Reviewer #1:** No comments.

**Reviewer #2:** No comments.

**Reviewer #3:**

- Please add the confidence interval (CI) measurement definition to the analysis section of the methods.

**Authors’ response:** Thank you very much for your constructive comment. We have added the confidence interval (CI) measurement definition in the manuscript on the analysis section.

- Some percentages were presented without decimals, some with one decimal, and some with two decimals. To maintain consistency, I suggest presenting all the percentages with one decimal.

**Authors’ response:** Thank you very much for your constructive comments. We have tried to make consistent all decimals throughout the document using one decimal.

- Line 151: Please correct "One hundred fourth one".

**Authors’ response:** Thank you very much for your constructive comment. We have corrected it in the manuscript.

- Line 166, suggested format: "83.1% (95 % confidence intervals (CIs): 78.9%, 86.6%)".

**Authors’ response:** Thank you very much for your constructive comment. We have corrected it as your suggestion.

- Until line 177, you used "," as a separator between confidence intervals, but after line 177 "-" was used instead. Please keep the consistency.

**Authors’ response:** Thank you very much for your critical constructive comment. We have used "-" throughout the document to make consistency.
